# Huang-Lian-Jie-Du Decoction Attenuates Atherosclerosis and Increases Plaque Stability in High-Fat Diet-Induced ApoE^-/-^ Mice by Inhibiting M1 Macrophage Polarization and Promoting M2 Macrophage Polarization

**DOI:** 10.3389/fphys.2021.666449

**Published:** 2021-09-02

**Authors:** Yinhe Cai, Junmao Wen, Siwen Ma, Zhexing Mai, Qunzhang Zhan, Yijun Wang, Yueyao Zhang, He Chen, Haiyi Li, Wei Wu, Rong Li, Chuanjin Luo

**Affiliations:** ^1^First Clinical Medical College, Guangzhou University of Chinese Medicine, Guangzhou, China; ^2^Department of Cardiovascular Medicine, First Affiliated Hospital of Guangzhou University of Chinese Medicine, Guangzhou, China

**Keywords:** Huang-Lian-Jie-Du decoction, atherosclerosis, plaque stability, macrophages polarization, ApoE^−/−^ mice

## Abstract

Macrophage polarization plays a vital impact in triggering atherosclerosis (AS) progression and regression. Huang-Lian-Jie-Du Decoction (HLJDD), a famous traditional Chinese decoction, displays notable anti-inflammatory and lipid-lowering effects in different animal models. However, its effects and mechanisms on AS have not been clearly defined. We determined whether HLJDD attenuated atherosclerosis and plaques vulnerability by regulating macrophage polarization in ApoE^−/−^ mice induced by high-fat diet (HFD). Furthermore, we investigated the effects of HLJDD on macrophage polarization in oxidized low-density lipoprotein (ox-LDL) induced RAW264.7 cells. For *in vivo* assay, compared with the model group, HLJDD ameliorated lipid metabolism, with significantly decreased levels of serum triglyceride, total cholesterol (CHOL), and lipid density lipoprotein. HLJDD suppressed serum tumor necrosis factor α (TNF-α) and IL-1β levels with increased serum IL-10 level, and inhibited mRNA level of NLRP3 inflammasome in carotid tissues. HLJDD enhanced carotid lesion stability by decreasing macrophage infiltration together with increased expression of collagen fibers and α-SMA. Moreover, HLJDD inhibited M1 macrophage polarization, which decreased the expression and mRNA levels of M1 markers [inducible nitric oxide synthase (iNOS) and CD86]. HLJDD enhanced alternatively activated macrophage (M2) activation, which increased the expression and mRNA levels of M2 markers (Arg-1 and CD163). For *in vitro* assay, HLJDD inhibited foam cell formation in RAW264.7 macrophages disturbed by ox-LDL. Besides, groups with ox-LDL plus HLJDD drug had a lower expression of CD86 and mRNA levels of iNOS, CD86, and IL-1β, but higher expression of CD163 and mRNA levels of Arg-1, CD163, and IL-10 than ox-LDL group. Collectively, our results revealed that HLJDD alleviated atherosclerosis and promoted plaque stability by suppressing M1 polarization and enhancing M2 polarization.

## Introduction

Atherosclerosis (AS) is recognized as the predominant pathological basis of cardiovascular diseases (CVDs; Frostegård, 2013; Bäck et al., 2019). Inflammatory processes and impaired lipid metabolism jointly contribute to the initiation and development of AS (Libby and Hansson, 2015; Mangge and Almer, 2019). Intimal macrophages lead to the maintenance of atherosclerotic inflammatory responses, and participate in the whole processes of AS (Barrett, 2020).

Numerous studies have emphasized the critical ability of macrophage polarization to trigger atherosclerosis progression and regression (Koelwyn et al., 2018; Shapouri-Moghaddam et al., 2018). Stimulated by different microenvironments such as oxidized lipids and cytokines, macrophage is polarized into M1 phenotype, classically activated, and M2 phenotype macrophage, alternatively activated (Mosser and Edwards, 2008; Sica and Mantovani, 2012). M1 polarized macrophages and subsequent releases of inflammatory cytokines such as inducible nitric oxide synthase (iNOS), interleukin 6 (IL-6), and tumor necrosis factor α (TNF-α) play a crucial mediator role in accelerating AS (Wang et al., 2014). In response to the cytokines IL-4 and IL-13, M2 macrophages are polarized and secrete anti-inflammatory factors such as IL-10 and collagen (Huang et al., 2014), which exerts anti-inflammatory properties and increases the stability of AS plaques (Rahman et al., 2017). Thus, inhibiting phenotypic conversion of macrophages to M1 and enhancing M2 enrichment may be a potential strategy to prevent and attenuate atherosclerosis.

From the perspective of traditional Chinese medicine (TCM), endogenous and exogenous heat and toxins are recognized as the pathogenic mechanisms of inflammation (Qi et al., 2019). Huang-Lian-Jie-Du decoction (HLJDD) is a representative prescription for dispelling heat and detoxicating, which is achieved by four crude herbs, Coptidis Rhizoma (*Coptis chinensis* Franch., Huang Lian), Scutellariae Radix (*Scutellaria baicalensis* Georgi., Huang Qin), Phellodendri Chinensis Cortex (*Phellodendron chinense* Schneid., Huang Bo), and Gardeniae Fructus (*Gardenia jasminoides* Ellis., Zhi Zi) in a weight ratio of 3:2:2:3. Currently, HLJDD has attracted accumulating interest owing to their superior biological activities in lipid-reducing (Jin et al., 2010; Zhang et al., 2014) and suppressing inflammatory responses (Li et al., 2014; Xu et al., 2017; Yuan et al., 2019). HLJDD significantly decreased the secretion of inflammatory cytokines and mediators stimulated by lipopolysaccharide (LPS) in RAW264.7 macrophages (Chen et al., 2016) or in high-fat diet (HFD) feeding ApoE^−/−^ mice (Ma et al., 2013). High Performance Liquid Chromatography (HPLC) analysis (Yuan et al., 2019) showed that HLJDD comprised lots of active ingredients, and the content of berberine, palmatine, baicalin, geniposide, coptisine, wogonoside, jatrorrhizine, and epiberberine accounted for 93.81% of the total content of 13 major characteristic constituents. Previous studies have demonstrated that multiple chemical ingredients isolated from HLJDD also exerted anti-atherosclerotic functions by diminishing macrophage infiltration and inhibiting inflammatory responses *in vivo* and *in vivo* (Zhang et al., 2017; Wu et al., 2019; Yu et al., 2020; Zhao et al., 2020; Supplementary Table S1). However, whether HLJDD might attenuate AS progression and plaque vulnerability by balancing macrophage polarization remain unclear. In the present study, we explored the role of HLJDD on macrophage polarization in ApoE^−/−^ mice fed a HFD and RAW264.7 macrophages induced by oxidized low-density lipoprotein (ox-LDL).

## Materials and Methods

### Preparation of HLJDD

Coptidis Rhizoma, Scutellariae Radix, Phellodendri Chinensis Cortex, and Gardeniae Fructus were crushed into small pieces and mixed in a ratio of 3:2:2:3. The mixture was soaked in pure water (1:10, w/v) for 1h, then boiled for 1.5h and the filtrates were collected. The residues were then refluxed in eight times purified water (v/w) for 1.5h, and then filtered. The two filtrates were combined and dried by rotary evaporation at 60°C. After further freeze-drying, HLJDD powder was obtained at a yield of 35.6% and stored at 4°C. Seven active components geniposide, baicalin, coptisine, epiberberine, berberine, phellodendrine, and palmatine in HLJDD extract were identified by Liquid Chromatograph Mass Spectrometer (LC-MS) as we previously reported (Li, 2019). Afterward, HLJDD powder was prepared with 0.5% carboxymethylcellulose sodium (CMC-Na) solution before the experiment.

### Atherosclerosis Animal Model Preparation and Treatment Procedure

The animal experiment was approved by the Animal Ethics Committee of Guangzhou University of Traditional Chinese Medicine (Permit number: 20191022007), and followed the Guide for the Care and Use of Laboratory Animals of the National Institutes of Health. Six-week-old male ApoE^−/−^ mice (specified pathogen free, 18±1g) were purchased from Guangzhou University of Traditional Chinese Medicine (Guangzhou, China, certificate no. 44005800007457). The animals were housed in specified pathogen free units of the Animal Center at Guangzhou University of Traditional Chinese Medicine, at 22±1°C, with a relative humidity of 60–70% and a 12h light/dark cycle. After 6weeks’ feeding of HFD (containing 15% lard, 0.5% sodium cholate, 2% vitelline, 1.5% CHOL, and 81% basic feed), the mice were randomly divided into five groups (10 mice per group): model, low dose HLJDD (HLJDD-L, 2.28g/kg), medium dose HLJDD (HLJDD-M, 4.55g/kg), high dose HLJDD (HLJDD-H, 9.1g/kg), and simvastatin (positive-control group, 3.0mg/kg) groups. The mice were orally gavage with 0.5% CMC-Na solution, various concentrations HLJDD or simvastatin once a day for 12weeks in combination with HFD feeding. Ten mice were fed with normal chow without any intervention as control group. The mice were administered intragastrically at a volume of 0.1ml/10g body weight.

The medical dosage of mice were 9.1 times that used in humans. In the present study, medium dose of HLJDD in mice was selected as the clinically equivalent dose of HLJDD in humans (HLJDD clinical dose: 30g/day, adult weight 60kg, the dose of mice was: 30g ÷ 60kg×9.1≈4.55g/kg). High and low doses of HLJDD were twice or 0.5 times of the equivalent doses in mice, respectively.

### Sample Collection

At the end of the experiment, the mice were anesthetized *via* 0.1% pentobarbital sodium and then blood samples were collected from the lateral caudal vein after fasting overnight. The serum was separated by centrifugation at 1000*g* for 10min at 4°C and stored at −80°C for further detection. Subsequently, the mice were sacrificed by dislocation, and the right common carotid arteries were detached. Then the carotid arteries of five mice in each group were fixed in 4% paraformaldehyde for histopathological examination. The remaining carotid arteries of the five mice were snap-frozen in liquid nitrogen for further tests.

### Analysis of Serum Lipid Concentrations

The serum supernatants were collected to determined lipid concentrations based on commercial kits using a biochemical autoanalyzer (URIT-8020A, China), including total CHOL, LDL cholesterol, and triglycerides (TG).

### Cytokines Detection by Enzyme-Linked Immunosorbent Assay

The contents of TNF-α, IL-1β, and IL-10 in serum were measured by ELISA kits (Cell Signaling Technology, United States) following the manufacturer’s instructions. The optical density (OD) values were recorded at 450nm within 5min using an ELASA autoanalyzer.

### Masson Trichrome Staining

Carotid tissues were fixed in 4% paraformaldehyde, then dehydrated, embedded in paraffin wax and sliced into 6-μm-thick sections. Cross-sections of carotid arteries were stained by masson trichrome staining according to standard histopathological methods. An optical microscope (Olympus, Japan) was used to observe and photograph the histopathological changes. The collagen contents in carotid arteries were analyzed by Image-Pro Plus 6.0 software.

### Immunohistochemical Staining

The carotid sections were immersed in antigen retrieval solution and dried in an oven at 60°C for 30min. The sections were then put into 3% H_2_O_2_ for 10min to block the endogenous peroxidase activity and incubated with normal goat serum for 15min at room temperature. Subsequently, primary antibodies were added in the sections separately at 4°C overnight in a humid chamber (anti-α-smooth muscle actin (α-SMA), ab5694, Abcam, United States; anti-Macrophage/Monocyte (MOMA-2), GTX39773, GeneTex, United States; anti-iNOS, ab115819, Abcam, United States; anti-arginine 1 (Arg-1), ab23354, Abcam, United States). After that, the sections were incubated with Biotinylated Goat anti-Mouse or Goat anti-Rabbit IgG (1:200) for 2h. After development with 3,30-diaminobenzidine (DAB) and counterstaining with hematoxylin, a light microscope was used to photograph the sections. Average optical density of staining area was semi-quantitatively calculated by Image-Pro Plus 6.0 software.

### Cell Culture

RAW264.7 macrophages were purchased form National Collection of Authenticated Cell Cultures (ID: TCM13). RAW264.7 cells were cultured in DMEM medium supplemented with 10% FBS and 1% penicillin-streptomycin, and maintained at 37°C in a humidified atmosphere with 5% CO_2_ for 24h. HLJDD lyophilized powder was dissolved in DMSO and then diluted to the required concentration with serum-free medium. The final concentration of DMSO was 0.01% (v/v).

### Cell Viability Assay

The cytotoxicity of HLJDD in RAW264.7 cells was evaluated with the CCK-8 assay. RAW264.7 macrophage cells (5×10^4^ cells/well) were seeded into 96-well plates and incubated with various concentrations of HLJDD (0, 5, 10, 20, 40, 80, and 160μg/ml) for 24h. CCK-8 (Beijing Labgic Technology Co., Ltd., China) solution (100μl/well) was then added to the cells, followed by cultivation for 1h. Then the absorbance of each well was measured under 450nm to detect cell viability.

### Oil Red O Staining *in vitro*

Briefly, *in vitro*, RAW264.7 cells were pretreated with HLJDD (20 or 40μg/ml) for 12h and then incubated with ox-LDL (80μg·ml^−1^) for 24h. Then the cells were washed three times in phosphate buffer saline (PBS; 0.01mol/L, PH=7.4) and fixed in 4% paraformaldehyde for 10min. Subsequently, the cells were stained with Oil Red O solution (0.5% in isopropanol, diluted with double distilled water (ddH2O) in ratio of 3:2) for 30min at 37°C and then washed with PBS again. Histopathological changes and cells with red-stained lipid droplets were observed and photographed using a stereomicroscope.

### Immunofluorescence Staining *in vitro*

Briefly, *in vitro*, RAW264.7 cells were seeded into 24-well plates and pretreated with HLJDD (20 or 40μg/ml) for 12h. The cells were then incubated with or without ox-LDL (80μg·ml^−1^) for 24h. Cells treated with PBS were used as the controls. Afterward, the RAW264.7 cells were fixed with 4% paraformaldehyde for 30min and transparent with 0.05% TritonX-100 for 10min. Subsequently, the specimens were washed with PBS and then blocked with 10% FBS to avoid the nonspecific fluorescence. Immunofluorescence staining was conducted with using primary antibodies against CD86 (1:200, ab112490, Abcam, United States) and CD163 (1:200, ab87099, Abcam, United States) at 4°C overnight. After that the samples were incubated with goat anti-rabbit IgG H&L, washed twice with PBS and stained with DAPI afterward. Photomicrographs were obtained using Zeiss Axiophot microscope and assessed by Image-Pro Plus 6.0 software.

### Quantitative Real-Time PCR *in vivo* and *in vitro*

*In vivo*, gene expression of NLRP3, iNOS, CD86, CD163, and Arg-1 at mRNA level within carotid homogenate supernatants of mice were detected. Briefly, total RNA was isolated and purified using RNApure extraction kit (BioTeke Corporation, Beijing, China) according to the manufacturer’s instructions. *In vitro*, RAW264.7 macrophages (5×10^4^ cells/well) were grown in 12-well plates. The cells were pretreated with HLJDD (20 or 40μg/ml) for 12h, and then exposed to ox-LDL (80μg·ml^−1^) for 24h. Cells treated with PBS were used as the controls. Afterward, the cell lysates using Trizol reagent (Invitrogen, United States) were extracted to determine the mRNA level of iNOS, CD86, IL-1β, Arg-1, CD163, and IL-10. The real-time PCR (RT-PCR) experiment was performed using the TB Green premix Ex Taq II (TaKaRa Biotechnology, Dalian, China) in CFX96 Real-Time PCR Detection System (Bio-Rad, United States). The specific primer sequences were listed as follows: NLRP3, forward primer 5'-ATTACCCGCCCGAGAAAGG-3' and reverse primer 5'-CATGAGTGTGGCTAGATCCAAG-3'; iNOS, forward primer 5'-GGATGCCGTAGGGAAGGAT G-3' and reverse primer 5'-GAGCAGCATTCGAGGGGAG-3'; CD86, forward primer 5'-TCAATGGGACTGCATATCTGCC-3' and reverse primer 5'-GCCAAAATACTACCAGCTCACT-3'; CD163, forward primer 5'-TGGCCTCTGAGTTTAGGGTCT-3' and reverse primer 5'-CCCTTGGTGTCGAACCAGC-3'; Arg-1, forward primer 5'-TTGGGTGGATGCTCACACTG-3' and reverse primer 5'-GTACACGATGTCTTTGGCAGA-3'; IL-1β, forward primer 5'-GAAATGCCACCTTTTGACAGTG-3' and reverse primer 5'-TGGATGCTCTCATCAGGACAG-3'; IL-10, forward primer 5'-TTGTCGCGTTTGCTCCCATT-3' and reverse primer 5'-GAAGGGCTTGGCAGTTCTG-3'. Fold levels in gene expression were determined by 2-^△△^Ct method. RT-PCR was conducted for three independent experiments or as indicated, using GAPDH as the reference gene.

### Statistical Analysis

All analyses were performed with SPSS Statistics 22.0 software. Data are presented as the mean±SD. Multigroup comparisons were determined by one-way ANOVA. Statistically significant difference was defined as *p*<0.05. All of the measurements were taken from distinct samples. Sample size is specified in the figure legends.

## Results

### HLJDD Enhances Carotid Lesion Plaques Stability

**Figure 1:**
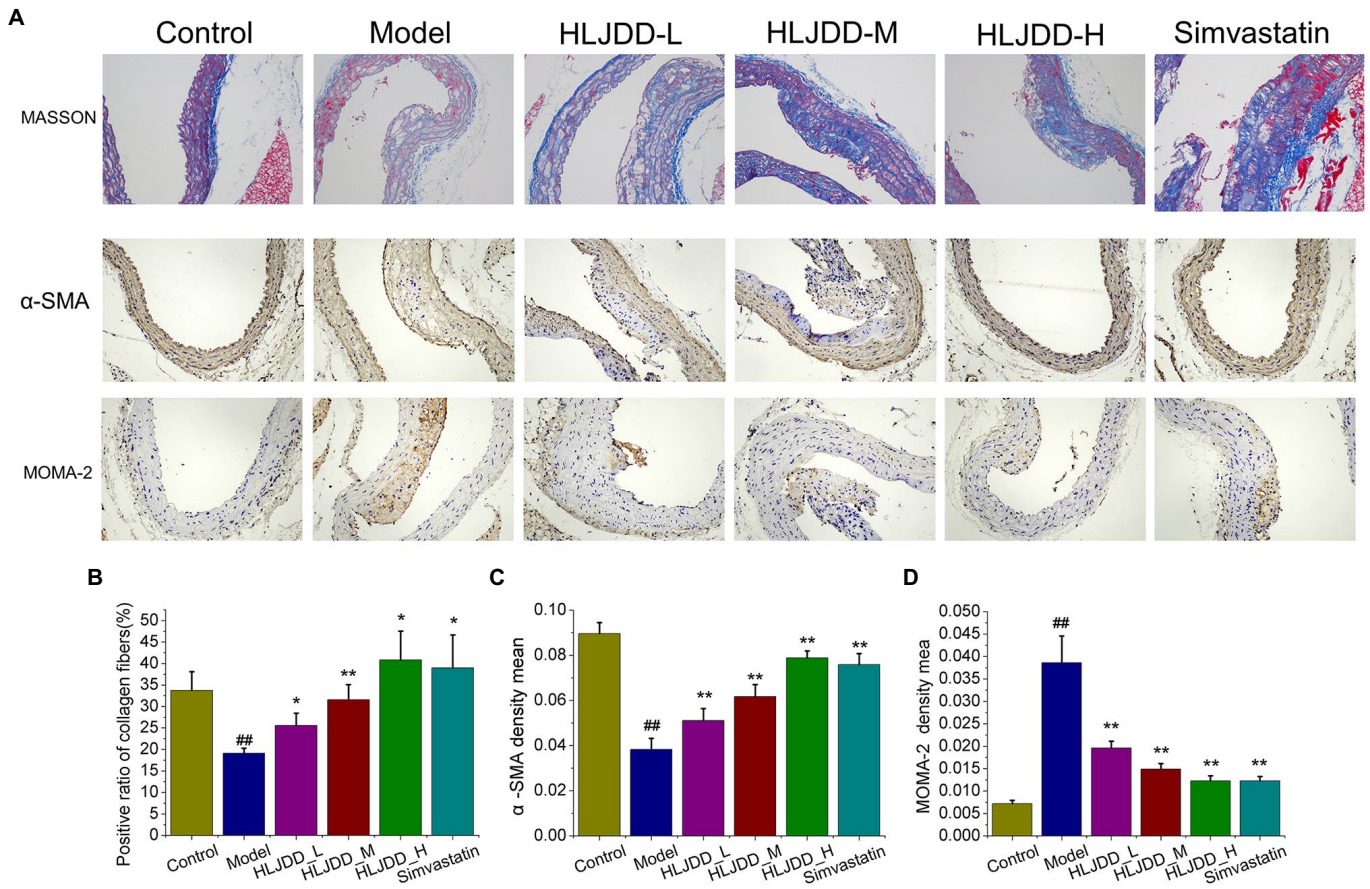
Effects of Huang-Lian-Jie-Du decoction (HLJDD) on plaques stability of carotid lesion in ApoE^−/−^ mice with atherosclerosis (AS). **(A)** Representative MASSON, immunohistochemical staining of α-SMA and MOMA-2 in carotid arteries sections are shown. Magnification, ×200; scale bar=200μm. **(B)** The positive radio of collagen content in carotid sections by masson trichrome staining. Values are expressed as the mean±SD. **(C)** The average optical density of α-SMA in carotid sections was semi quantitatively analyzed by Image Pro Plus 6.0 software. Values are expressed as the mean±SD. **(D)** The average optical density (OD) of MOMA-2 in carotid sections was semi quantitatively analyzed by Image Pro Plus 6.0 software. Values are expressed as the mean±SD (*n*=5; ^#^*p<*0.05 and ^##^*p<*0.01 vs. the control group; ^*^p<0.05 and ^**^p<0.01 vs. the model group; one-way ANOVA).

We explored the protective effects of HLJDD treatment on plaque compositions. The results of histopathological examination of carotid tissues indicated that the typical presence of atherosclerotic plaques, subintimal adipocytes and thickened artery walls of the model group was observed in the carotid arteries (Figure 1A). Compared with the control group, collagen content in arterial walls of the model group, highlighted in blue by masson staining, decreased significantly (*p<*0.05; Figure 1B). However, these changes could be attenuated by the treatment with HLJDD. Collagen content in arterial walls was effectively increased after HLJDD of medium (*p<*0.01) and high dosage (*p<*0.05) or simvastatin treatment (*p<*0.05) compared with the model group (Figure 1B), which contributed to plaque stabilization. Since vascular smooth muscle cells (VSMCs) are the main maker of collagen and proteoglycans in the fibrous matrix, plaque stability is directly correlated with the amount of VSMCs (Bennett et al., 2016). The content of α-SMA in carotid arteries, one of the strongly express differentiated VSMCs markers (Chappell et al., 2016), could be substantially increased by HLJDD at high dosage (*p<*0.01) or simvastatin (*p<*0.01) compared with those in model mice, as determined by immunostaining with α-SMA (Figures 1A,C). As shown in Figure 1A, after 18weeks on a high-fat diet, apparent MOMA2 positive content was observed in carotid lesions of the model mice. HLJDD treated groups (*p<*0.01) or simvastatin group (*p<*0.01) could significantly decrease local infiltration of monocytes/macrophages compared with model group (Figure 1D). Taken together, these data indicated that HLJDD could retard carotid atherosclerotic plaque progression and enhance plaque stability.

### HLJDD Attenuates Serum Lipid Profiles and Inflammatory Responses in Mice

**Figure 2:**
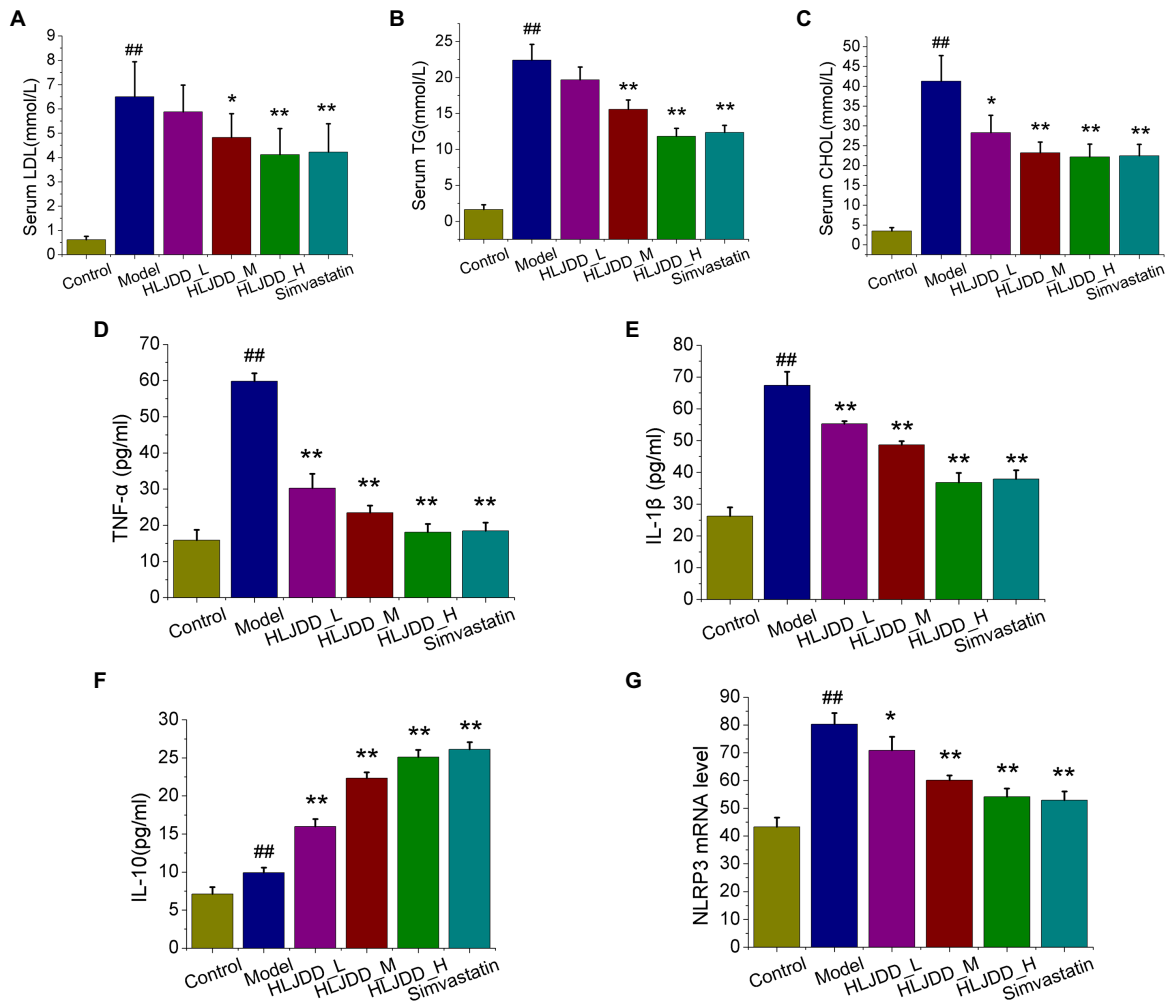
Effects of HLJDD on serum lipid profiles and inflammatory cytokines in ApoE^−/−^ mice with AS. The effects of HLJDD on low density lipoproteins (LDL) levels **(A)**, triglyceride (TG) levels **(B)**, and total cholesterol (CHOL) levels **(C)** of serum samples are shown. The effects of HLJDD on serum levels of tumor necrosis factor α (TNF-α; **D**), IL-1β **(E)**, and IL-10 **(F)** by ELISA are shown. The effect of HLJDD on NLRP3 mRNA expression **(G)** in carotid tissues is shown. Values are expressed as the mean±SD (*n*=5; ^##^*p<*0.01 vs. the control group; ^*^p<0.05 and ^**^p<0.01 vs. the model group; one-way ANOVA).

Atherogenic lipoproteins are ingested and accumulated in macrophages, which give rise to the subsequent inflammation and formation of foam cells (Moore et al., 2018; Bäck et al., 2019). Firstly, LDL, TG, and CHOL of serum samples were detected. As results, concentrations of LDL, TG, and CHOL in model group were higher than those in control group (*p<*0.01). Compared with those in model group, HLJDD treatment at medium (4.55g/kg) and high (9.1g/kg) dosage could significantly decrease serum TG (*p<*0.01) and LDL (*p<*0.01) parameters, as well as 3.0mg/kg simvastatin (*p<*0.01; Figures 2A,B). And all HLJDD administrated groups showed a significant decrease in serum CHOL compared with model group (*p<*0.05 or *p<*0.01; Figure 2C).

Inflammation is recognized as a critical role in promoting plaque formation and progression, we further explored three typical serum inflammatory cytokines TNF-α, IL-1β, and IL-10 by ELISA method. After 18weeks of the feeding experiment, the serum levels of IL-6, TNF-α, and IL-10 in model group increased compared with those in control group (*p*<0.01), and all the intervention group significantly relieved the elevated levels of pro-inflammatory cytokines TNF-α (*p<*0.01) and IL-1β (*p<*0.05 or *p<*0.01) as compared to model group (Figures 2D,E). Moreover, significant serum level increase in IL-10 was obtained for HLJDD at high and medium dosage or simvastatin compared with those in model group (Figure 2F). Interestingly, the mRNA level of NLRP3 inflammasome in carotid tissues, a link between lipid metabolism and plaque inflammation (Sica and Mantovani, 2012; Libby and Hansson, 2015), exhibited distinguished dose-independent decrease in HLJDD-treated groups (*p<*0.05, *p<*0.01, *p<*0.01) compared with that in model mice (Figure 2G). These results suggested that HLJDD administration has the potential to treat AS.

### HLJDD Suppresses M1 Polarization and Promotes M2 Polarization in Mice

**Figure 3:**
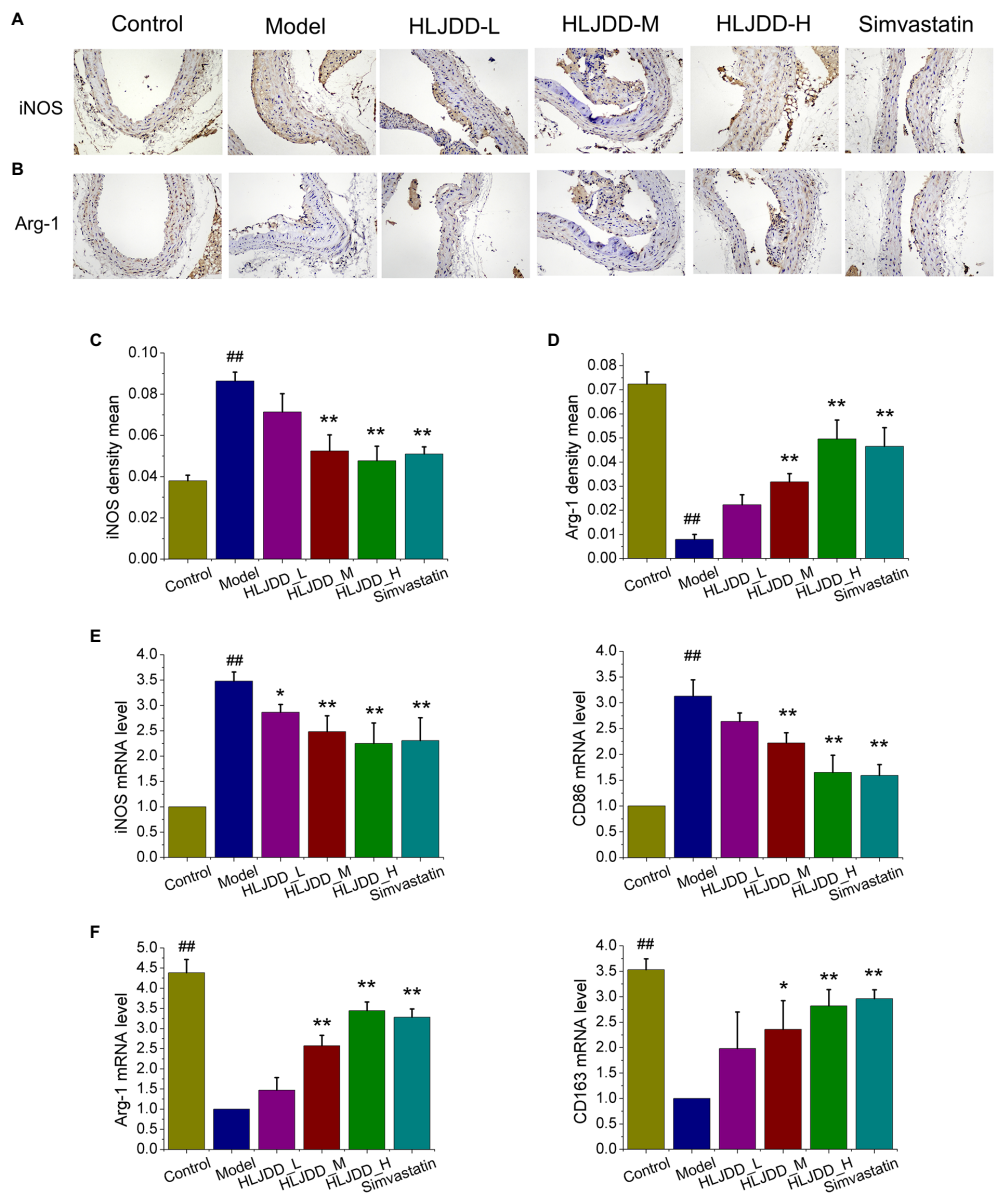
Effects of HLJDD on macrophage polarization in ApoE^−/−^ mice with AS. **(A)** Representative immunohistochemical staining showed the expression of inducible nitric oxide synthase (iNOS) in carotid sections. Magnification, ×200; scale bar=200μm. **(B)** Representative immunohistochemical staining showed the expression of Arg-1 in carotid sections. Magnification, ×200; scale bar=200μm. **(C)** The average optical density of iNOS in carotid sections was semi quantitatively analyzed by Image Pro Plus 6.0 software. Values are expressed as the mean±SD (*n*=5; ^##^*p*<0.01 vs. the control group; ^*^p<0.05 and ^**^p<0.01 vs. the model group; one-way ANOVA). **(D)** The average density of Arg-1 in carotid sections was semi quantitatively analyzed by Image Pro Plus 6.0 software. Values are expressed as the mean±SD (*n*=5; ^*^p<0.05 and ^**^p<0.01 vs. the model group; one-way ANOVA). **(E)** The mRNA expression of iNOS and CD86 in carotid tissues were detected by real-time PCR (RT-PCR; *n*=5; ^##^*p*<0.01 vs. the control group; ^*^p<0.05 and ^**^p<0.01 vs. the control group; one-way ANOVA). **(F)** The mRNA expression of Arg-1 and CD163 in carotid tissues were detected by RT-PCR. Values are expressed as the mean±SD (*n*=5; ^##^*p*<0.01 vs. the model group; ^*^p<0.05 and ^**^p<0.01 vs. the model group; one-way ANOVA).

Macrophage polarization plays a vital role in the formation and progression of atherosclerotic lesions (Koelwyn et al., 2018; Shapouri-Moghaddam et al., 2018). Therefore, we detected the phenotypes of macrophage in atherosclerotic lesions to determine whether HLJDD attenuates inflammation and vulnerable plaque formation by regulating macrophage polarization. As shown in Figure 3A, the content of iNOS, M1 macrophage-associated marker, in carotid plaques of model group increased significantly (*p<*0.01) compared with control group, and it exhibited an obvious decrease in HLJDD at medium (*p<*0.01) and high dosage (*p<*0.01) or 3.0mg/kg simvastatin (*p<*0.01) compared with model group (Figure 3C). After conducting RT-PCR with carotid tissues, the mRNA levels of M1 markers iNOS and CD86 (Figure 3E) were increased in model group compared with control group, but it could be strikingly decreased by HLJDD treated groups or simvastatin treated group.

The enrichment of M2 macrophages has been determined to promote atherosclerotic lesion repair and plaque stability (Rahman and Fisher, 2018). As shown in Figure 3B, the expression of M2 macrophage-associated marker Arg-1 in carotid plaques of model group decreased significantly compared with those in control mice, as detected by immunohistochemical staining. Compared with the model group, the medium (*p<*0.01) and high (*p<*0.01) dosage of HLJDD or 3.0mg/kg simvastatin (*p<*0.01) exhibited a significant increase on the expression of Arg-1 in carotid plaques (Figure 3D). Moreover, we also explored the mRNA levels of M2 macrophage marker. As shown in Figure 3F, the mRNA levels of Arg-1 and CD163 were strikingly increased in medium (*p<*0.01, *p<*0.01, *p<*0.05) and high (*p<*0.01) dose of HLJDD treated groups or 3.0mg/kg simvastatin (*p<*0.01) treated group compared with those in model mice.

### Effects of HLJDD on RAW264.7 Cells Viability

**Figure 4:**
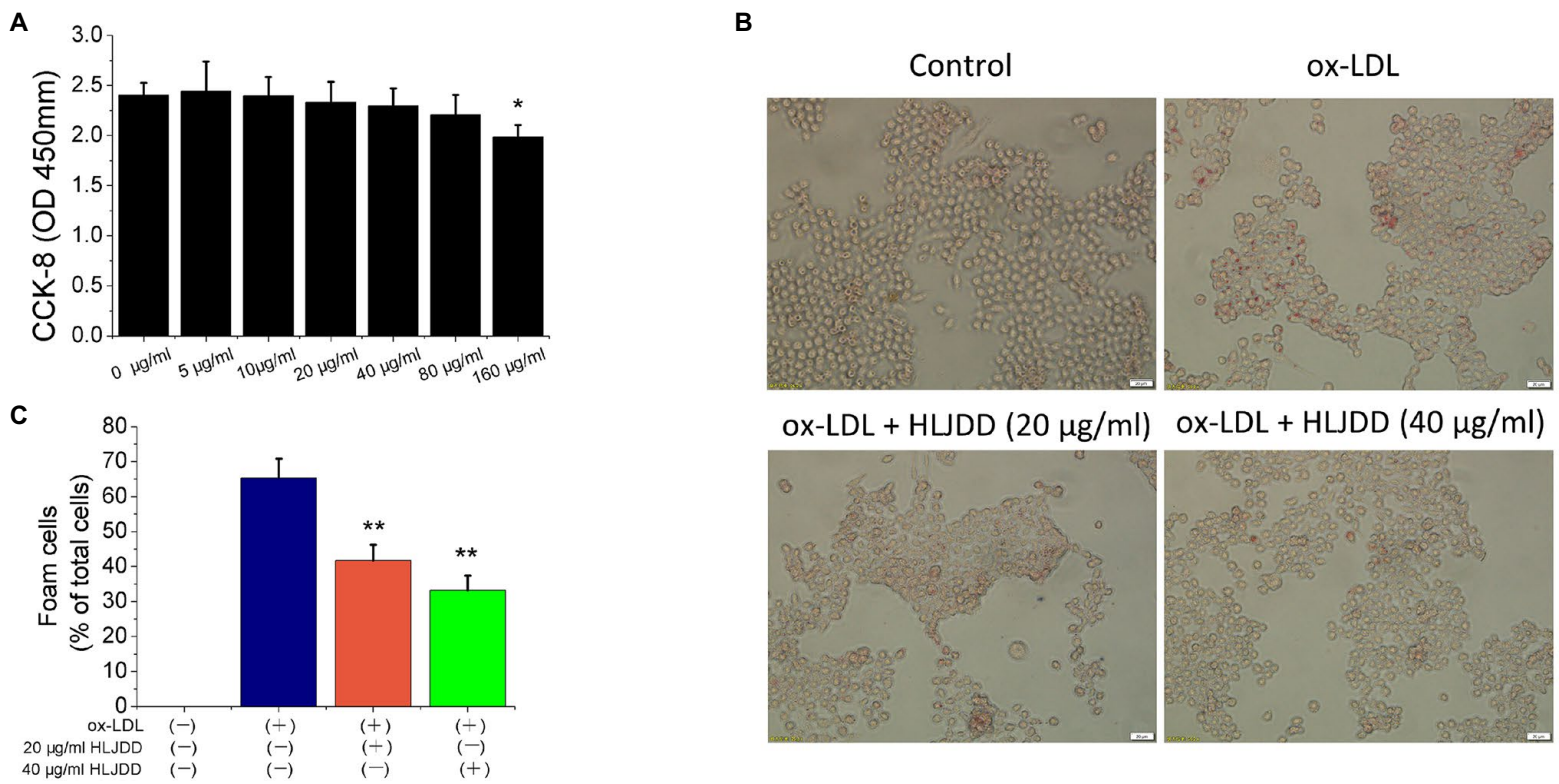
Huang-Lian-Jie-Du decoction inhibited ox-LDL-induced RAW264.7 cells lipid accumulation. **(A)** Cytotoxicity of HLJDD on RAW264.7 cells. Values are expressed as the mean±SD from three independent experiments (^**^p<0.01 vs. with control group). **(B)** Representative images of Oil Red O staining *in vitro* RAW264.7 cells. Scale bar=20μm. **(C)** Percent positive foam cells were measured by Image J software. Values are expressed as the mean±SD from three independent experiments (^**^p<0.01 vs. ox-LDL-treated group; one-way ANOVA). ox-LDL, oxidized low-density lipoprotein.

In ApoE^−/−^ mice experiments, we found that HLJDD alleviated AS and promoted plaque stability by modulating macrophage polarization. Therefore, we tried to verify the anti-atherogenic mechanism of HLJDD *in vitro*. CCK-8 assay was used to detect the survival rate of RAW264.7 macrophages at different concentrations of HLJDD (0–160μg/ml). The results showed that HLJDD had no cytotoxic effect on RAW264.7 cells when HLJDD was less than 80μg/ml (Figure 4A). Therefore, the concentration of HLJDD at 20 and 40μg/ml was selected for subsequent experiments.

### HLJDD Ameliorated Foam Cell Formation Induced by ox-LDL in RAW264.7 Macrophages

Macrophage-derived foam cell formation was induced by ox-LDL and ultimately leads to plaques instability. Therefore, we used 80μg/ml ox-LDL to induce the formation of foam cells *in vitro* experiment. As shown in Figure 4B, lipid accumulation was increased by ox-LDL, but both 20 and 40μg/ml HLJDD obviously reduced the cytoplasmic lipid droplet accumulation induced by ox-LDL in RAW 264.7 cells (Figure 4C). These results indicated that HLJDD may inhibit ox-LDL induced foam cell formation in RAW264.7 macrophages.

### HLJDD Shifts M1/M2 Polarization *in vitro*

**Figure 5:**
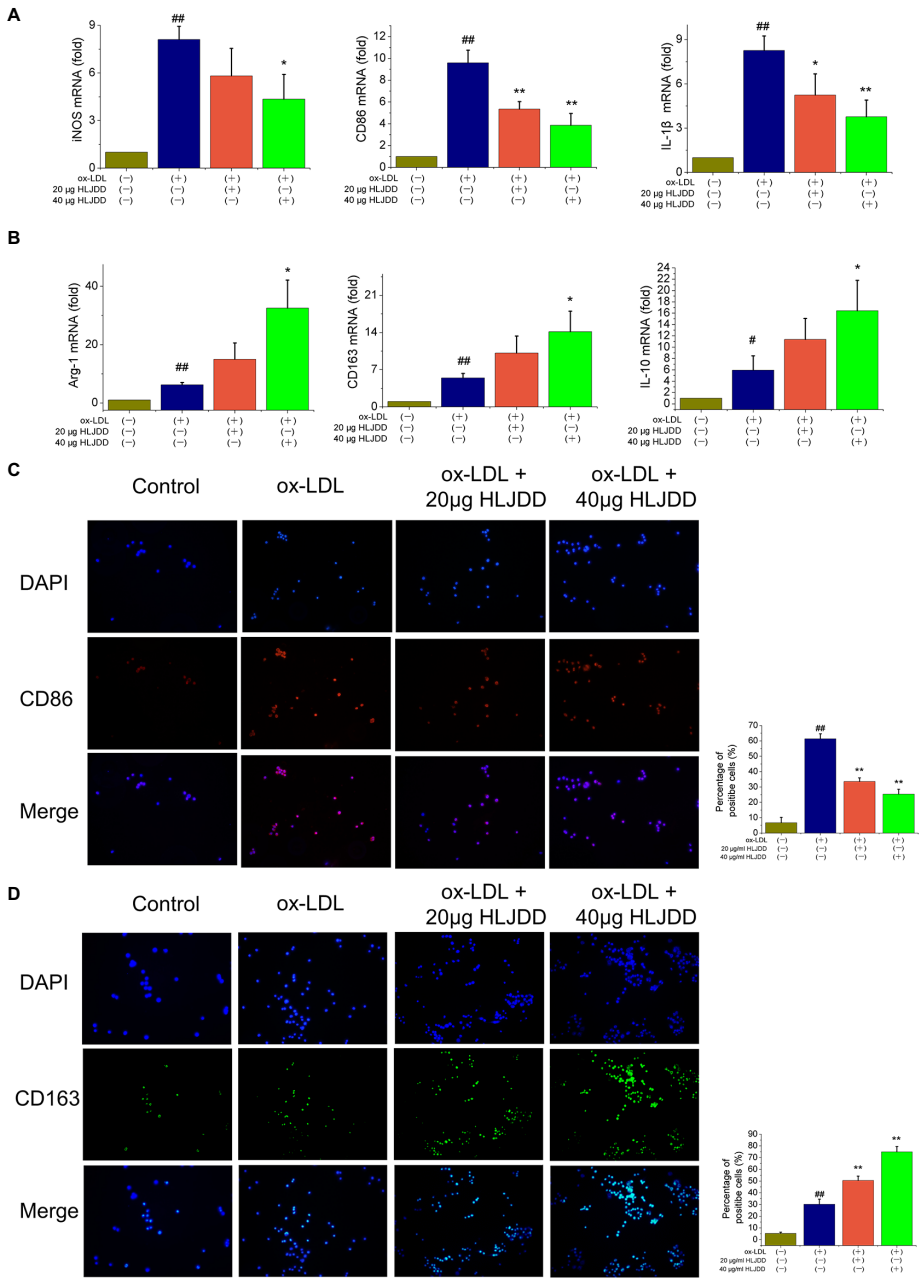
Effects of HLJDD on macrophage polarization in ox-LDL-induced RAW264.7 cells. RAW264.7 cells were pretreated with HLJDD (20 or 40μg/ml) for 12h, and then exposed to ox-LDL for another 24h. **(A)** The mRNA level of iNOS, CD86, and IL-1β were detected by real-time PCR. Values are expressed as the mean±SD. **(B)** The mRNA level of Arg-1, CD163, and IL-10 were detected by real-time PCR. Values are expressed as the mean±SD. **(C)** Representative immunofluorescence images showing CD86 expression in RAW264.7 cells. The percentage of cells that were positive for CD86 fluorescence were quantified by Image Pro Plus 6.0 software. **(D)** Representative immunofluorescence images showing CD163 expression in RAW264.7 cells. The percentage of cells that were positive for CD163 fluorescence were quantified by Image Pro Plus 6.0 software. Values are expressed as the mean±SD from three independent experiments (^##^*p<*0.01 vs. the control group; ^*^p<0.05 and ^**^p<0.01 vs. the model group; one-way ANOVA).

To further verify the effects of HLJDD on macrophage polarization, an *in vitro* model was established using cultured RAW264.7 cells. As shown as Figure 5A, mRNA levels of M1 markers such as iNOS, CD86, and IL-1β increased after ox-LDL stimulation, and the results above were inhibited by HLJDD treatment. About 40μg/ml HLJDD showed a much stronger inhibition. Moreover, immunofluorescence result of CD86 also demonstrated that HLJDD inhibited ox-LDL-induced CD86 secretion (Figure 5C).

Besides, as shown in Figure 5B, ox-LDL-stimulated RAW264.7 macrophage cells expressed decreased mRNA levels of M2 markers such as Arg-1, CD163, and IL-10. Importantly, these effects could be further enhanced by HLJDD treatment. Immunofluorescence on CD163 showed that the inhibiting effect of ox-LDL on CD163 expression could also be enhanced by HLJDD treatment (Figure 5D).

Taken together, these observations suggested that HLJDD could downregulate the phenotypic conversion of macrophage to M1 and enhance M2 polarization both in ApoE^−/−^ mice and in RAW264.7 macrophages cells.

## Discussion

Although, lipid-reducing and anti-inflammatory drugs have been effectively used to treat atherosclerosis, the dose-dependent adverse effects associated with rhabdomyolysis and glucose homeostasis prompted us to find effective and less toxic new drug candidates (Collins et al., 2016; Ward et al., 2019). In this study, we demonstrated that treatment with HLJDD, a TCM formula, attenuated atherosclerosis and promoted plaque stability in HFD-induced AS ApoE^−/−^ mice by modulating macrophage polarization.

Currently, the regulation of M1/M2 phenotypic macrophage balance is an emerging therapeutic target for various inflammation-based disorders (Sica and Mantovani, 2012; Lutgens et al., 2019), and it plays a critical impact on inflammatory responses and lesion formation in AS. Stimulated by various microenvironments, lesion macrophages can be polarized as either pro-inflammatory M1, classically activated, or anti-inflammatory M2, alternatively activated (Sica and Mantovani, 2012; Moore et al., 2013). M1 macrophages secrete pro-inflammatory factors and are enriched in rupture-prone and unstable lesions, while M2 macrophages are abundant in regressing lesions and promote plaque stability. This study indicated that HLJDD treatment blocked HFD-driven inflammation and vulnerability of atheroma plaques in ApoE^−/−^ mice, which was related to the capacity of HLJDD to suppress M1 polarization and enhance M2 polarization. An *in vitro* experiment also indicated that HLJDD could alter the M1/M2 polarization in RAW264.7 macrophages cells stimulated by ox-LDL.

Impaired cholesterol metabolism in arterial macrophages has conventionally been known to cause AS (Sica and Mantovani, 2012). In this context, atherogenic lipoproteins such as ox-LDL that accumulate in arteries are ingested by macrophages, and give rise to the subsequent formation of foam cells and their production of inflammatory cytokines, which contribute to plaque progression and destabilization (Moore et al., 2013). In our study, we found that HLJDD decreased ox-LDL-induced foam cell formation in RAW264.7 macrophages through classical Oil red O staining. Moreover, consistent with the lipid-reducing effect in previous reports *via* HFD induced hyperlipidemia rats (Jin et al., 2010), or HFD and streptozotocin induced Type 2 diabetic rats (Zhang et al., 2014), HLJDD still reduced serum TG, LDL, and CHOL in high-fat diet feeding ApoE^−/−^ mice, and the lipid-reducing effects of HLJDD were in a dose-dependent manner. The above data showed a superior lipid-lowering and antiatherogenic effects in HLJDD, and it was a therapeutic potential as a prodrug candidate for AS treatment.

Cholesterol crystals and ox-LDL that accumulating in atherosclerotic plaques are proposed to activate inflammasomes such as NLRP3 inflammasome in local macrophages, resulting in inflammatory cytokines IL-1β and IL-18 production (Duewell et al., 2010; Guo et al., 2015). Silence of NLRP3 inflammasome has been reported to suppress AS and maintains the stabilization of plaque in ApoE^−/−^ mice (Zheng et al., 2014). In our study, we observed that HLJDD administration significantly decreased the mRNA level of NLRP3 inflammasome in carotid tissues compared with those in model mice. Interestingly, the inhibition of representative compounds isolated from HLJDD on NLRP3 activation was previously demonstrated *in vivo* and vitro. Berberine (Zhu et al., 2018) or Coptisine (Wu et al., 2019), the representative alkaloids isolated from HLJDD, suppressed IL-1β secretion and NLRP3 inflammasome activation in ox-LDL stimulated macrophages. Baicalin (Zhao et al., 2020), representative flavonoids isolated from HLJDD, also exerted similar inhibitory effects in ApoE^−/−^ mice. These results have suggested that HLJDD contains various ingredients, and antagonizes local activation of NLRP3 inflammasome caused by hyperlipidemia.

Atherosclerosis is a progressive inflammatory process (Ross, 1999), and the joint actions of inflammatory processes and lipid metabolism have been recognized as a causal factor in forming atherosclerotic plaques (Hansson and Hermansson, 2011; Libby and Hansson, 2015). Previous studies *in vivo* and *in vitro* have emphasized the anti-inflammatory effect of HLJDD in many inflammatory conditions. Chen et al. (2016) indicated that HLJDD inhibited the LPS-stimulated secretion of inflammatory mediators IL-1β, IL-4, and MCP-1 in RAW264.7 cells *via* the NF-κB and MAPKs inactivation. An experiment in acute ulcerative colitis mouse model showed that HLJDD significantly decreased plasma TNF-α and IL-1β levels *via* NF-κB signaling pathway (Yuan et al., 2019). In this study, we found that compared with that feeding high-fat diet for 18weeks, cofeeding with HLJDD dose-dependently suppressed serum levels of pro-inflammatory cytokines IL-1β and TNF-α expression in ApoE^−/−^ mice. Interestingly, IL-1β is a NLRP3 inflammasome activation product that play a vital role in atherogenesis, which can also upregulate some inflammatory components such as TNF-α and IL-8 to jointly intensify inflammation and plaque formation (Hoseini et al., 2018). Monoclonal antibodies against IL-1β is able to inhibit atherosclerotic plaque formation *in vivo* (Kirii et al., 2003; Bhaskar et al., 2011). These observations point out that HLJDD might be a potential strategy for the treatment of AS.

M1 macrophages were polarized in response to oxidized lipoproteins and cytokines, and then accelerated plaque progression and vulnerability by releasing pro-inflammatory cytokines including high levels of IL-1β, IL-6, and TNF-α (Mosser and Edwards, 2008; Barrett, 2020). In our study, we observed a dose-dependent decreased secretion of iNOS and mRNA levels of M1 markers iNOS and CD86 in carotid tissues after HLJDD consumption. Furthermore, HLJDD also significantly restrained M1 macrophage polarization in ox-LDL induced RAW264.7 cells. Taken together, HLJDD treatment exerted anti-inflammatory and anti-atherosclerotic properties in a dose-dependent manner, which may be related to its inhibition on M1 polarization and NLRP3 inflammasome activation.

Atherosclerotic plaques with increased vulnerability are prone to rupture, and subsequent formation of thrombus leads to severe cardiovascular events, accounting for about 50% of deaths worldwide (Frostegård, 2013; Libby et al., 2019; Yap et al., 2020). At present, enhancing stability of plaques and preventing expansion of vulnerable plaques have become the focus of current treatment for AS which has already formed. Of note, composition of plaques, such as the proportion of collagen, VSMCs, extracellular lipid, or macrophages, has been recognized as the determinant of lesion stability (Naghavi et al., 2003; Wolf and Ley, 2019). On the one hand, strong evidences have observed that plaques could be destabilized by a thin fibrous cap due to its few VSMCs and collagen content, which are always prone to ruptured (Lim and Park, 2014). Our study found that HLJDD treatment upregulated the amounts of collagen fibers in carotid arteries according to masson staining. Moreover, the expression of α-SMA, as one of the strongly express differentiated VSMCs markers of the fibrous cap (Chappell et al., 2016), was also improved by the administration of HLJDD. On the other hand, in each vascular bed, plaque macrophages give rise to the maintenance of the local inflammatory responses and promote the progression of plaques into complicated, rupture-prone lesions, and its inhibition also has been recognized as a therapeutic target in stemming the progression of AS and stabilizing existing lesions (Barrett, 2020). As expected, we found that HLJDD administration dose-dependently reduced monocytes and macrophages infiltration in local lesions of HFD fed ApoE^−/−^ mice. Interestingly, macrophage efferocytosis is considered as a protective anti-inflammatory capacity of M2 macrophages (Kojima et al., 2016; Yurdagul et al., 2017) to clear apoptotic cells and debris, thereby to reduce necrotic core formation in atherosclerotic plaques (Moore et al., 2018; Bäck et al., 2019). Thus, it may be a viable strategy to promote AS regression and plaque stabilization with the enrichment of M2 macrophages or enhancement of macrophage efferocytotic function (Chinetti-Gbaguidi et al., 2011). Our study observed an increased expression of M2 markers and IL-10 level after HLJDD consumption compared with those in model mice. Moreover, an *in vitro* experiment also showed that HLJDD significantly evoked mRNA level of M2 markers Arg-1, CD163 and IL-10 secretion and the expression of CD163 in ox-LDL induced RAW264.7 macrophages, which were in accordance with the *in vivo* results. Interestingly, increased IL-10 expression might be involved in promoting the regulatory T (Treg) cell responses and M2_C_ efferocytosis during inflammation resolution (Han and Boisvert, 2015). Taken together, the inhibition of HLJDD decreased macrophage infiltration, which, together with the increased expression of collagen fibers and α-SMA, might further stabilize the vulnerability of atheroma plaques in atherosclerotic ApoE^−/−^ mice, and it may be closely correlated with the enhancement on M2 polarization and macrophage efferocytosis of HLJDD treatment.

## Conclusion

**Figure 6:**
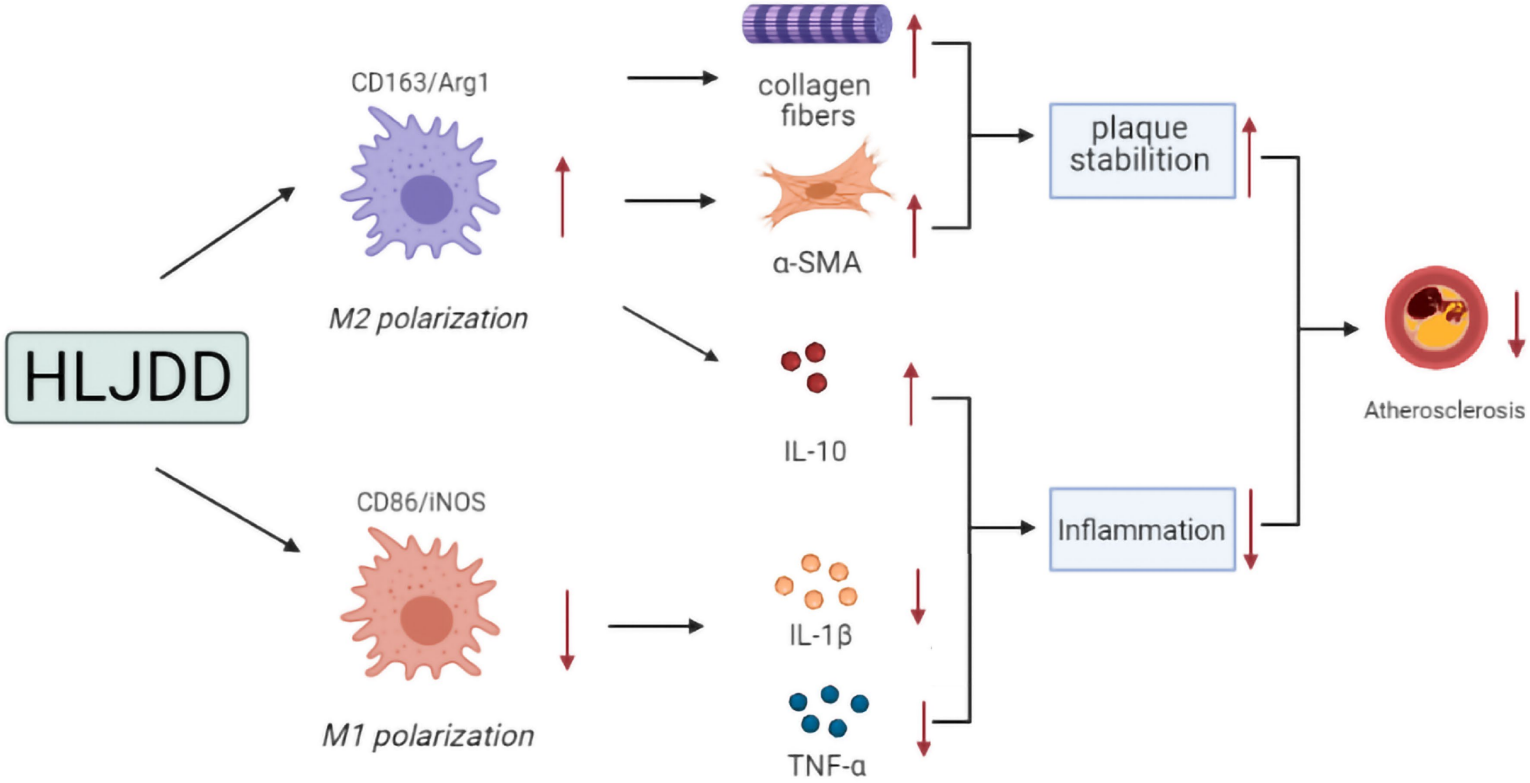
Possible mechanism pathway of the anti-atherosclerotic effect of HLJDD.

Our findings suggested that HLJDD treatment might be a potential drug for atherosclerosis with attenuation of atherosclerotic progression and enhancement of plaque stability. These effects followed a dose-dependent manner and seemed to be mediated by macrophage polarization, with high-dose HLJDD treatment exerting the most pronounced effects. Possible mechanisms are shown in Figure 6.

## Data Availability Statement

The raw data supporting the conclusions of this article will be made available by the authors, without undue reservation.

## Ethics Statement

The animal study was reviewed and approved by the Animal Ethics Committee of Guangzhou University of Traditional Chinese Medicine (permit number: 20191022007).

## Author Contributions

JW and YC: hypothesis conception and design. CL, RL, and WW: experiments instructors. JW, ZM, and YC: experiments performers. YC, QZ, SM, and HC: data acquisition and analysis. YC, JW, SM, HL, YZ, and YW: manuscript drafters. All authors contributed to the article and approved the submitted version.

## Conflict of Interest

The authors declare that the research was conducted in the absence of any commercial or financial relationships that could be construed as a potential conflict of interest.

## Publisher’s Note

All claims expressed in this article are solely those of the authors and do not necessarily represent those of their affiliated organizations, or those of the publisher, the editors and the reviewers. Any product that may be evaluated in this article, or claim that may be made by its manufacturer, is not guaranteed or endorsed by the publisher.
