# Supplementary material for: Huang-Lian-Jie-Du Decoction Attenuates Atherosclerosis and Increases Plaque Stability in High-Fat Diet-Induced ApoE-/- Mice by Inhibiting M1 Macrophage Polarization and Promoting M2 Macrophage Polarization
Source: Front Physiol. 2021 Sep 2;12:666449. doi: 10.3389/fphys.2021.666449 (PMC8445160; doi:10.3389/fphys.2021.666449)
Supplement: Supplementary file 1 [file Table_1.docx]

Table S1. Anti-atherosclerotic actions and therapeutic or regulatory mechanisms of representative activated compounds isolated from HLJDD

| **Representative activated compounds** | **Model** | **Mechanisms** | Refs. |
| --- | --- | --- | --- |
| **Geniposide** | Lysophosphatidic acid (LPA) stimulated RAW264.7 macrophages  High-fat diet-induced AS ApoE^-/-^ mice | inhibiting the formation of foam cells via p38/MAPK and AKT signaling pathways  Ameliorating disrupted lipid profiles and the development of atherosclerosis | Shen D, Zhao D, Yang X, Zhang J, He H, Yu C. Geniposide against atherosclerosis by inhibiting the formation of foam cell and lowering reverse lipid transport via p38/MAPK signaling pathways. Eur J Pharmacol. 2019 Dec 1;864:172728. doi: 10.1016/j.ejphar.2019.172728 |
|  | High-fat diet-induced AS New Zealand rabbits | Inhibiting inflammatory responses and stabilizing atherosclerotic plaques via modulating macrophage polarization | Jin Z, Li J, Pi J, Chu Q, Wei W, Du Z, Qing L, Zhao X, Wu W. Geniposide alleviates atherosclerosis by regulating macrophage polarization via the FOS/MAPK signaling pathway. Biomed Pharmacother. 2020 May;125:110015. doi: 10.1016/j.biopha.2020.110015. |
|  | LPS-stimulated RAW264.7 macrophages  High-fat diet-induced AS ApoE^-/-^ mice | Inhibiting inflammatory responses via miR-101/ MKP-1/p38 signaling pathway | Cheng S, Zhou F, Xu Y, et al. Geniposide regulates the miR-101/MKP-1/p38 pathway and alleviates atherosclerosis inflammatory injury in ApoE-/- mice. Immunobiology. 2019;224(2):296-306. doi:10.1016/j.imbio.2018.12.005 |
| **Berberine** | High-fat diet-induced AS ApoE^-/-^ mice | Improving serum lipid and systemic inflammation levels, and alleviating atherosclerosis by modulating gut microbiota | Wu M, Yang S, Wang S, et al. Effect of Berberine on Atherosclerosis and Gut Microbiota Modulation and Their Correlation in High-Fat Diet-Fed ApoE-/- Mice. Front Pharmacol. 2020;11:223. Published 2020 Mar 13. doi:10.3389/fphar.2020.00223  [Zhu L, Zhang D, Zhu H, et al. Berberine treatment increases Akkermansia in the gut and improves high-fat diet-induced atherosclerosis in Apoe-/- mice. Atherosclerosis. 2018;268:117-126. doi:10.1016/j.atherosclerosis.2017.11.023] |
|  | Macrophage-derived foam cells | Inhibiting oxLDL uptake and promoting cholesterol efflux in macrophage foam cell via suppressing AP-1 Activity and Activation of the Nrf2/HO-1 Pathway | Yang XJ, Liu F, Feng N, et al. Berberine Attenuates Cholesterol Accumulation in Macrophage Foam Cells by Suppressing AP-1 Activity and Activation of the Nrf2/HO-1 Pathway. J Cardiovasc Pharmacol. 2020;75(1):45-53. doi:10.1097/FJC.0000000000000769 |
|  | Western-type diet diet-induced AS ApoE^-/-^ mice | Reducing serum lipid levels, alleviating atherosclerotic lesions, and reducing oxidative injury | Tan W, Wang Y, Wang K, et al. Improvement of Endothelial Dysfunction of Berberine in Atherosclerotic Mice and Mechanism Exploring through TMT-Based Proteomics. Oxid Med Cell Longev. 2020;2020:8683404. Published 2020 May 31. doi:10.1155/2020/8683404 |
| Palmatine | High-fat diet-induced AS hamsters | Ameliorating disrupted lipid profiles by up-regulating LDLR and CYP7A1, down-regulating ASBT, as well as enhancing fecal excretion of TC and TBA | Ning N, He K, Wang Y, et al. Hypolipidemic Effect and Mechanism of Palmatine from Coptis chinensis in Hamsters Fed High-Fat diet. Phytother Res. 2015;29(5):668-673. doi:10.1002/ptr.5295 |
| **Baicalin** | Oxidized low‑density lipoprotein (ox‑LDL)‑treated human aorta vascular smooth muscle cells (HA‑VSMCs) | inhibiting proliferation and promotes apoptosis of vascular smooth muscle cells by regulating the MEG3/p53 pathway | Liu Y, Jia L, Min D, Xu Y, Zhu J, Sun Z. Baicalin inhibits proliferation and promotes apoptosis of vascular smooth muscle cells by regulating the MEG3/p53 pathway following treatment with ox‑LDL. Int J Mol Med. 2019;43(2):901-913. doi:10.3892/ijmm.2018.4009 |
|  | High-lipid diet-induced AS ApoE^-/-^ mice | Relieving oxidative stress and inflammatory responses via inactivating NF-κB and p38 MAPK signaling pathways | Wu Y, Wang F, Fan L, et al. Baicalin alleviates atherosclerosis by relieving oxidative stress and inflammatory responses via inactivating the NF-κB and p38 MAPK signaling pathways. Biomed Pharmacother. 2018;97:1673-1679. doi:10.1016/j.biopha.2017.12.024 |
|  | High-fat diet-induced AS ApoE^-/-^ mice | Inhibiting oxidative stress and inflammatory responses via suppressing NLRP3 inflammasome | Zhao J, Wang Z, Yuan Z, Lv S, Su Q. Baicalin ameliorates atherosclerosis by inhibiting NLRP3 inflammasome in apolipoprotein E-deficient mice. Diab Vasc Dis Res. 2020;17(6):1479164120977441. doi:10.1177/1479164120977441 |
| **Coptisine** | High fat and high cholesterol (HFHC) induced obese Syrian golden hamsters | Ameliorating disrupted lipid profiles and inflammatory responses by LPS/TLR-4-mediated signaling pathway | Zou ZY, Hu YR, Ma H, et al. Coptisine attenuates obesity-related inflammation through LPS/TLR-4-mediated signaling pathway in Syrian golden hamsters. Fitoterapia. 2015;105:139-146. doi:10.1016/j.fitote.2015.06.005 |
|  | High-fat diet-induced AS ApoE^-/-^ mice | Ameliorating disrupted lipid profiles and inflammatory responses by MAPK/NF-κB-dependent pathway | Feng M, Kong SZ, Wang ZX, et al. The protective effect of coptisine on experimental atherosclerosis ApoE-/- mice is mediated by MAPK/NF-κB-dependent pathway. Biomed Pharmacother. 2017;93:721-729. doi:10.1016/j.biopha.2017.07.002 |
| **Jatrorrhizine** | Normal diet feeding Syrian golden hamsters | Improving the utilization and excretion of cholesterol by up-regulating the mRNA and protein expression of LDLR and CYP7A1. | Wu H, He K, Wang Y, et al. The antihypercholesterolemic effect of jatrorrhizine isolated from Rhizoma Coptidis. Phytomedicine. 2014;21(11):1373-1381. doi:10.1016/j.phymed.2014.05.002 |
|  | An obesity mouse model | Ameliorating disrupted lipid profiles via the suppression of lipogenesis and the enhancement of lipid oxidation in the liver | Yang W, She L, Yu K, et al. Jatrorrhizine hydrochloride attenuates hyperlipidemia in a high-fat diet-induced obesity mouse model. Mol Med Rep. 2016;14(4):3277-3284. doi:10.3892/mmr.2016.5634 |
| Epiberberine | High fat and high cholesterol (HFHC) induced dyslipidemia Syrian golden hamsters | Inhibiting the synthesis of cholesterol, promoting the uptake and conversion of TC in liver and increasing the excretion of TC and TBA in feces | Zou ZY, Hu YR, Ma H, Feng M, Li XG, Ye XL. Epiberberine reduces serum cholesterol in diet-induced dyslipidemia Syrian golden hamsters via network pathways involving cholesterol metabolism. Eur J Pharmacol. 2016;774:1-9. doi:10.1016/j.ejphar.2015.11.017 |
